# Manufacturing of a Compact Micro Air Bearing Device for Power Micro Electro Mechanical System (MEMS) Applications Using Silica Film Assisted Processing

**DOI:** 10.3390/mi9040166

**Published:** 2018-04-02

**Authors:** Mingxing Yu, Pin Lv, Tiantong Xu, Xiao Tan, Haiwang Li

**Affiliations:** 1 School of Energy and Power Engineering, Beihang University, Beijing 100191, China; yumingxing1314@buaa.edu.cn (M.Y.); l1p2@buaa.edu.cn (P.L.); christxtt@163.com or tiantong@mit.edu (T.X.); tanxiao_buaa@foxmail.com or by1404143@buaa.edu.cn (X.T.); 2 National Key Laboratory of Science and Technology on Aero Engines Aero-Thermodynamics, Beijing 100191, China; 3 The Collaborative Innovation Center for Advanced Aero-Engine of China, Beijing 100191, China

**Keywords:** micro air bearing, silicon dioxide film, DRIE etch, wet etch, bonding, power MEMS

## Abstract

The focus of this study is on the manufacturing of micro air bearings (MABs) using silica film assisted processing. Structure of the three-layer micro air bearing is described in detail and the salient process flow of etching and bonding is illustrated. The main manufacturing challenges and the methods adopted to overcome them are also presented. The uniformity of wet etching for nozzles with 20 μm in diameter to silica film is improved by adopting an ultrasound assisted method. Particular attention is given to the novel fabrication procedures for the second layer of MAB (with three depths on aft side). This paper develops new applications of silica film in Micro Electro Mechanical System (MEMS) processing for MABs to realize the etching of multi-depth on the same side and efficient three-layer bonding with increased bonding areas. A silica etch mask is proven to achieve a higher accuracy in surface topography when compared to a photoresist mask for multi-depth etching, resulting in precise depth and vertical control. The bonding rate of three-layer direct bonding for MAB is increased by 50% from 0.05 to 0.1 with the novel silica film protection method.

## 1. Introduction

Micro turbine engine is a typical power Micro Electro Mechanical System (MEMS) system [1]. Due to its high energy density and small volume weight, it has important applications in micro air vehicles (MAVs), portable energy, and other extensive fields. To realize output of high energy density, the micro rotor (D ≈ 10 μm) is required to rotate stably in a limited space at ultra-high speed (~10^5^ rpm). Together with low friction resistance and compact structure, micro air bearings (MABs) have become the best choice for supporting micro rotors. Great achievements have been made in MABs and their lubrication characteristics [2], damping stiffness analysis [3,4], nonlinear dynamic characteristics [5,6,7], performance testing [8,9,10,11], and more. Nevertheless, only a few publications have mentioned the manufacturing process of MABs.

To obtain a high-performance MAB, high-accuracy machining is essential and significant. The published manufacturing methods mainly focus on deep reactive ion etching (DRIE) and the fusion-bonding of silicon wafers. Lin et al. [12] first presented the process flow of a five-wafer stack device based on fusion-bonding, and Luc et al. [13] improved its fabrication process. The process parameters that resulted in bowed and tapered journal bearings were investigated by Shan et al. [14] to improve the profile of journal bearings and micro journal bearings to obtain a sidewall verticality of almost 90°. M. Boufnichel et al. [15] studied the defects of bow and the considered ions were the factors that were causing passivation layer damage. Arturo A. et al. [16] discussed contacting and annealing procedures to produce a bonded wafer without etched structures. The reported research mainly focused on etching and bonding processes that form three-dimensional (3-D) structures directly, while less attention was paid to the pre-process before them.

Many pre-processes have a great impact on quality of etching and bonding in the practical fabrication of MABs, in which formation and application of silica film are of great importance and interest. The difficulties are mainly distributed in the uniformity control of the wet etching to silica film and its application in multi-depth etching and multi-layer silicon direct bonding (SDB). Haixin Zhu et al. [17] investigated deep fused silica etching using different masking methods and single-coated silicon-based thin film was demonstrated to be the best masking material. All micro-wells in Zhu’s study were 100 microns in diameter, which were larger than the nozzles of MAB in this study and had less trouble in bubble problems. Multi-layer SDB is a key process for the assembly of MABs. The structure of MABs is usually designed compact to reduce the number of bonding layers, which causes many separated trench structures with different depths of cross-sectional profile in both sides of a wafer. Teo C.J. [18] adopted a nested mask approach to fabricate feature structure with two depths on the same side of a wafer using a one-step thermal oxidation process, where a different masking material was used for each etch and all of the masking materials (photoresist and oxide) were patterned prior to any DRIE process. Etching to trenches with two-depth in the same side was also achieved based on the nested mask method [19,20,21,22] using a two-step thermal oxidation process. Separated feature structures with three depths can be fabricated by using a three-step thermal oxidation process based on these methods, but many cycles of thermal oxidation would significantly enhance the chances of wafer breakage and increase residual stress, which made the etched wafers hard to be bonded. Therefore, the fabrication of separated feature structures with three depths on the same side by using a one-step thermal oxidation process is still an urgent problem to be solved.

Microfabrication methods of the MAB using silica film assisted processing are investigated in this paper. First, the structure and the key microfabrication process flows for the constituent wafers that comprise the three-wafer stack micro air bearing are presented. Next, the main microfabrication challenges and the strategies that are adopted to overcome them are outlined. The wet etching process is improved by using an ultrasound assisted method to increase the uniformity of nozzles for thrust bearing applications. Attention is given to achieve separated feature structures with three depths on the aft side of the second layer by using a one-step thermal oxidation process. The DRIE etching experiment for multi-depth structure is conducted with a silica mask and a photoresist mask, respectively. The morphology control level of the etched structure is obtained by microscope. A comparative experiment of three-layer SDB is conducted focusing on the impact of silica film protection on the bonding rate of wafers. An infrared camera and a MATLAB image processing program are used for the qualitative and quantitative detection of bonding rates, respectively.

## 2. Structure and Microfabrication Process Flow

### 2.1. Structure and Components of MAB

**Figure 1:**
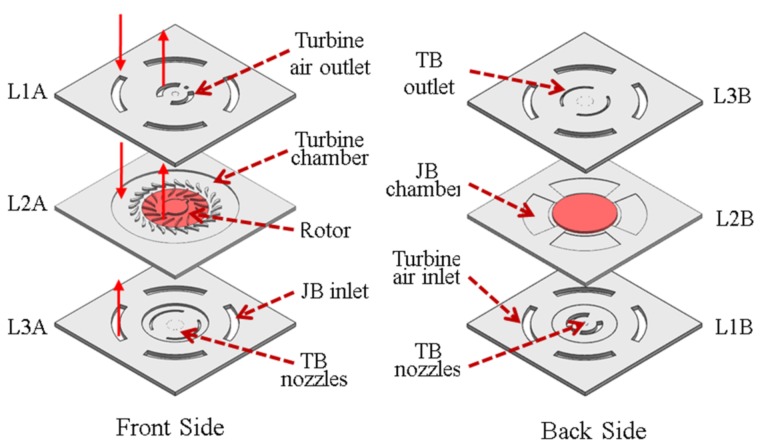
Exploded diagram of the micro air bearing rig.

The MAB with an overall size of 2 × 2 × 1.5 mm consists of three layers. An exploded view showing the individual layers of the device is presented in Figure 1. The rotor is located in the second and third wafer and is supported by two different air bearings in axial and radial directions. Nozzles arrays with 20 μm-diameter and 200 μm-depth are arranged in L1 layer and L3 layer, respectively, to provide the pair of hydrostatic thrust bearings (TB). A hydrostatic journal bearing (JB) is designed to support the bearing radially by clearance of only 10 microns in depth. The L1 layer consists of turbine air inlet channels, turbine air outlet channels, forward thrust bearing nozzles, and a port for fiber-optic sensor. The L2 layer comprises the turbine chamber, journal bearing chamber, stator blade, journal bearing, and rotor. The L3 layer consists of the journal bearing inlet channel, aft thrust bearing nozzles, and thrust bearing outlet.

**Figure 2:**
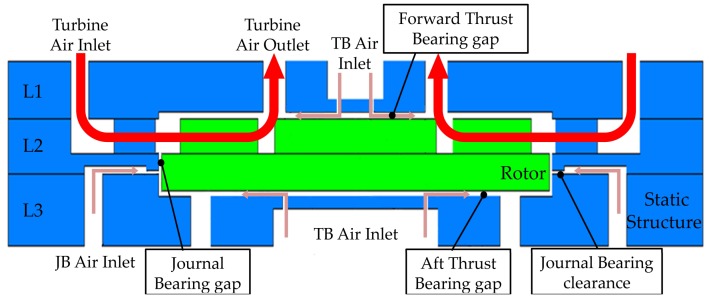
Cross section diagram of a bonded stack.

Cross section diagram of a bonded MAB is shown in Figure 2. Turbine driven air is supplied through the turbine air inlet channel and is driven to the turbine chamber in the second layer to rotate the rotor. The JB air is supplied from the JB air inlet in the third layer and then enters the JB chamber to form a high-pressure gas film between the stator and the rotor after being pressurized by JB clearance of 10 μm in depth, thus providing journal support for the micro rotor. TB airflow is divided into two parts and is driven into the TB nozzles in the first layer and the third layer, respectively, to provide axial support for the micro rotors by a gas film formed between rotor and stator.

### 2.2. Microfabrication Process Flow

**Figure 3:**
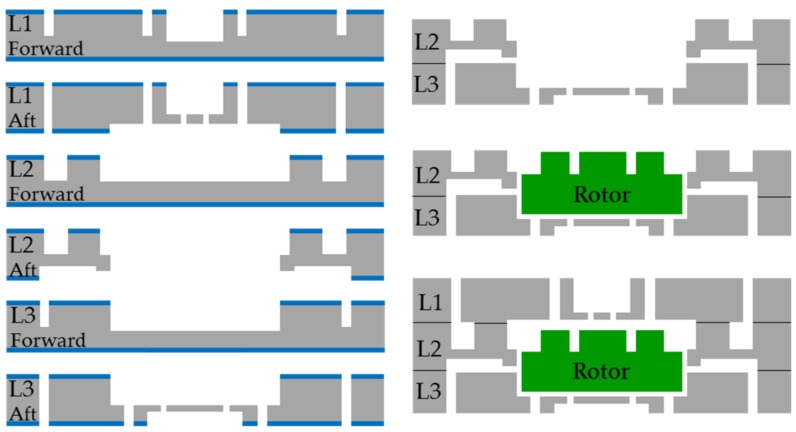
Key microfabrication process flow for micro air bearings (MAB).

The salient steps in the process flow are illustrated in Figure 3. Four-inch, P-type, double-sided polished (DSP) silicon wafers (provided by Suzhou Crystal Silicon Electronic & Technology Co., Ltd., Suzhou, China) with 500 μm thickness, 20–40 Ω·m resistivity, and 2 μm-thick thermal oxidation silica film are used in the fabrication process of MABs. Ambient temperature and humidity of the clean room are controlled strictly at 23–25 °C and 45–50%, respectively. The fabrication starts by etching the shallow global alignment marks on both sides of all the wafers with the help of a BG-406S double-side alignment exposure machine. Next, on the forward side of the L1 layer, two patterns are etched to depths of 250 μm and 300 μm, which define a turbine air inlet (outlet) and TB plenum. Then, on the aft side of the L1 layer, three patterns are etched to depths of 250 μm, 10 μm, and 200 μm as a turbine air inlet (outlet), TB gap and TB air inlet nozzles, respectively. Accordingly, the blades and rotor space are etched to depths of 250 μm by a deep reactive ion etching (DRIE) process on the forward side of the L2 layer. The turbine chamber, JB clearance, and rotor space are etched to depths of 100 μm, 10 μm, and 250 μm on the opposite side of the L2 layer. Subsequently, on the forward side of the L3 layer, the JB air inlet, and rotor space are etched to depths of 300 μm. Two patterns are etched to depths of 200 μm and 100 μm to define the JB air inlet, TB air outlet, TB air inlet nozzles, and TB plenum. The rotors with a total thickness of 800 μm and 250 μm high blade are entirely fabricated from one single layer. Following the etching process, all the wafers are cleaned by acetone and piranha solution (H_2_SO_4_:H_2_O_2_ = 3:1, volume ratio) to remove photoresist and is followed by a HF solution (HF:DI water = 1:10, volume ratio) etch of oxide. Once the oxide is removed, the wafers are dried by N_2_. The bonding process starts with the L3 layer and L2 layer. These two layers are loaded in the chamber of AML AWM-04 bonder to achieve alignment and surface contact under ambient temperature. Then, the rotors are put into their space and the L1 layer is aligned and contacted with the L2 layer. Next, the wafer stack is heated from ambient temperature to 100 °C, 150 °C, 200 °C, 250 °C, and 300 °C successively with each heating process taking 4 min. Once each specified temperature is reached, it begins to reheat again. The stack is held at 300 °C for 30 min to increase bond strength. During this heating process, the bonding force is held at 1500 N by the force roller. The bonded wafer stack is then loaded into a quartz boat and placed in a 1100 °C annealing chamber for 2 h. Following annealing, the bonded wafer stack is diced into 2 cm × 2 cm slices completing the micro bearing rig fabrication process.

## 3. Key Microfabrication Methods

### 3.1. BOE Etching of Oxide

**Figure 4:**
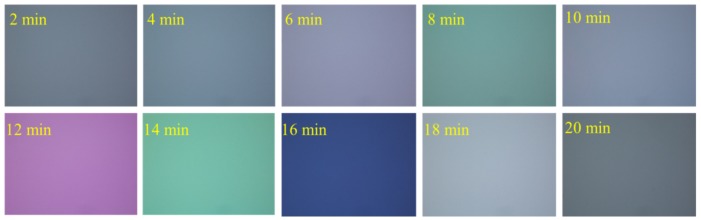
Apparent color of a silica film at different etching time in Buffered Oxide Etch (BOE) solution.

A BOE (Buffered Oxide Etch) solution (49% HF:40% NH_4_F = 1:6, volume ratio) was used to etch silica film that was formed by the thermal oxidation method. The temperature of the solution approximately equaled the ambient temperature during the whole process. To obtain an etching rate for a BOE solution to silica film, the thickness of silica film was tested by Think Focus CL2-MG70 confocal displacement sensor (Sixian Photoelectric Technology Co., Ltd., Shanghai, China) every two minutes. This time interval was chosen to form enough change of thickness and color, which can be detected accurately by the sensor and observed by Nikon MM-400 microscope. Furthermore, the wafer was observed under the microscope to record the color of the silica film, which was then compared with a color chart for silica, as shown in Figure 4. The results show that the etch rate of silica film in BOE solution is 100 nm/min, which provides a reference etch rate and helps to avoid superfluous and insufficient wet etching.

**Figure 5:**
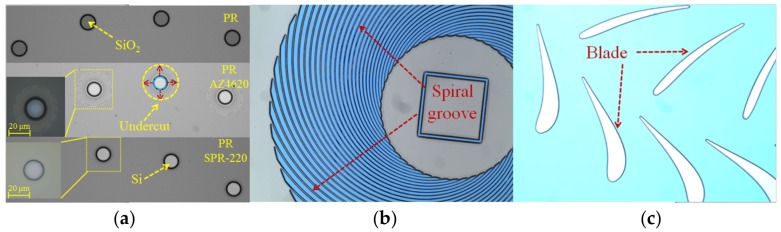
Wet etch result of typical structure in MABs with different masking and etching methods. (**a**) 20 μm-diameter nozzles wet etched by traditional and novel method; and, (**b**,**c**) Patterns of spiral groove and blade etched using novel method.

Nozzle arrays in the first and the third layers were designed to provide axial support for the micro rotor. Three key steps were required to fabricate nozzles: exposure and development, BOE etch of silica film, and DRIE etch of silicon. Adhesion promoter RZN6000 was first spin-coated prior to the deposition of AZ4620 photoresist to improve the adhesion between surface of Si and photoresist. A nozzle pattern with a clear edge was initially observed after exposure and development, as shown as the first part of Figure 5a. However, severe undercut occurred during the next 40-min BOE wet etching process around poor adhesion areas. Meanwhile, other regions were not completely etched due to bubbles, as shown in the second part of Figure 5a. Initial selectivity of Si DRIE process with respect to the resist is 75:1, but it would reduce to 50:1 after 200 μm-depth DRIE for nozzles because of higher temperatures on wafer that were caused by the longer etching process. Therefore, if AZ4620 photoresist was used as etching mask, 6–8 μm thickness was needed to etch nozzles with 200 μm in depth. However, 6–8 μm thickness and poor adhesion with oxide made it easy to gather little bubbles inside the silica holes (20 μm in diameter) during the wet etching process. As mentioned above, the etch rate of silica film in BOE solution was 100 nm/min and it took 20 min for a full etching of 2 μm-thick silica film at least. The bubbles gathered in the holes obstruct the chemical reaction between the BOE solution and silica film, thus causing longer and inhomogeneous etching. Therefore, a novel method aimed at improving the uniformity of BOE etching to silica film was proposed to increase adhesion and expel bubbles. A thinner photoresist SPR220 (4–6 μm) with better adhesion and an ultrasonic method for expelling bubbles were applied in the wet etching process. To avoid the reduction of adhesion by excessive ultrasound, ultrasonic vibration with power of 240 W was implemented for two seconds per minute. The third part in Figure 5a shows that nozzle pattern has been transferred from the photoresist to the silica film uniformly without undercutting. The same wet etch methods were also used in the common structures of MABs, such as spiral grooves and blades, where both integrity and uniformity were improved, as shown in Figure 5b,c).

**Figure 6:**
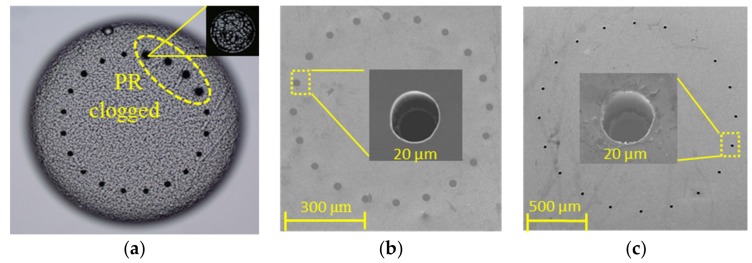
Nozzles etched using different mask methods. (**a**) Nozzles clogged using AZ4620 as a deep reactive ion etching (DRIE) mask; (**b**,**c**) Etched nozzles with the silica mask method in L1 (**b**) and L3 (**c**) layer, respectively.

The nozzles etched under the mask of the AZ4620 photoresist is shown in Figure 6a and some nozzles were clogged by photoresist, and thus uniformity of the arrays was poor. On the contrary, Figure 6b,c show the etched nozzles using SPR220 and oxide as wet etching mask and DRIE etching mask, respectively. Additionally, ultrasonic vibration was adopted in the wet etching process. The uniformity and the integrity of etched nozzles were greatly improved in the L1 layer and L3 layer with the new mask method, as shown in Figure 6b,c.

### 3.2. DRIE Etching of Si

**Figure 7:**
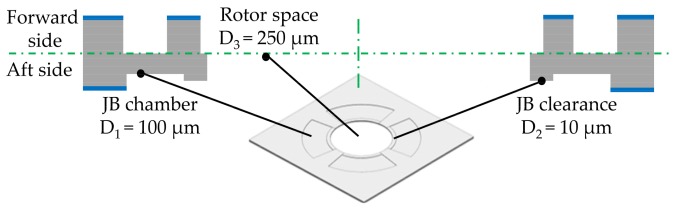
Cross section view of L2 layer with three depths on aft side.

**Figure 8:**
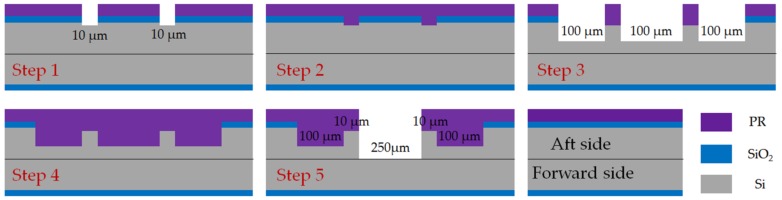
Process flow of traditional method for DRIE multi-depth structures on aft side of the second layer in MABs.

**Figure 9:**
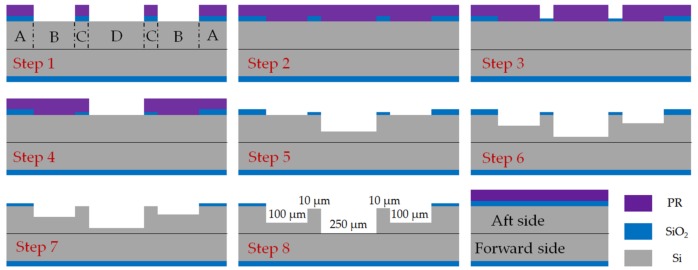
Process flow of traditional method for DRIE multi-depth structures on aft side of the second layer in MABs.

To achieve a compact structure, separated structures with different depths were designed on the same side of every layer. As shown in Figure 7, three trench structures with different depths (D_1_ = 100 μm, D_2_ = 10 μm, D_3_ = 250 μm) that were fabricated on aft side of the second layer would be used as rotor space, journal bearing clearance, and journal bearing chamber, respectively. Two methods were proposed to obtain multi-depth structures by DRIE etching and morphology of the etched structure was detected through a Nikon MM-400 microscope and SNE 4500 SEM. The traditional method only used photoresist as an etching mask (shown in Figure 8) and the innovative method first proposed in this paper took both 2 μm-thick oxide and photoresist as etching mask (shown in Figure 9). When compared with the nested mask methods proposed by Teo, C.J. and Shan, X.C. [18,19,20,21,22], the applied process sequence in this paper only needs one oxidation process to achieve three depths, and oxidation can be done before any process by wafer manufacturer, which means there will not be any further oxidation during the whole fabrication process in lab.

Traditional Method: the SPR220 photoresist was coated on the surface with silicon at a rotating speed of 6000 rpm to guarantee 6 μm thickness. Soft bake was 115 °C for 90 s and post exposure baking was 120 °C for 90 s. Exposure intensity was 10 mW/cm^2^ for 6 s and was developed by NMD-3 (2.38% Tetramethylammonium Hydroxide) for 3 min. The key process flow was illustrated, as follows:

Step 1 pattern, BOE etch oxide and DRIE for 10 μm JB clearance.
Step 2 remove photoresist and recoating 6 μm fresh photoresist.
Step 3 pattern, BOE etch oxide and DRIE for 100 μm JB chamber and rotor space.
Step 4 remove photoresist and recoating 6 μm fresh photoresist.
Step 5 pattern and DRIE for 250 μm rotor space.

It took three photoresist coating steps, two BOE steps and three DRIE steps to fabricate separated structures three depths on the aft side of the L2 layer. As shown in Figure 8, it is significant that in the third coating step, a 6 μm-thick photoresist was required to cover a 100 μm-depth trench that was fabricated in the second DRIE step, which was inadequate to achieve uniform coverage.

Innovative method: the process parameters of coating, exposure, development, and etching were the same as the traditional method. As shown in Figure 9, the key microfabrication process flow includes:

Step 1 pattern, BOE etch 2 μm oxide for JB chamber and rotor space.
Step 2 remove photoresist and recoating 6 μm fresh photoresist.
Step 3 pattern, BOE etch 1 μm oxide for JB clearance.
Step 4 remove photoresist, recoating 6 μm fresh photoresist, pattern for rotor space.
Step 5 DRIE for 150 μm rotor space, remove photoresist.
Step 6 DRIE for 90 μm JB chamber and rotor space.
Step 7 BOE etch 1 μm oxide to reveal oxide mask for JB clearance.
Step 8 DRIE for 10 μm JB clearance, JB chamber and rotor space.

It also took three coating steps, but the difference lies in the second and third coating steps. Using the innovative method, the second and third coating steps were arranged after the first BOE etching step, thus the 6 μm-thick photoresist only needed to cover 2 μm-deep oxide, rather than a 100 μm-deep silicon structure. Depths of Si and silica at each structure after each etching are shown in Table 1 to illustrate the change in depths in detail.

**Figure 10:**
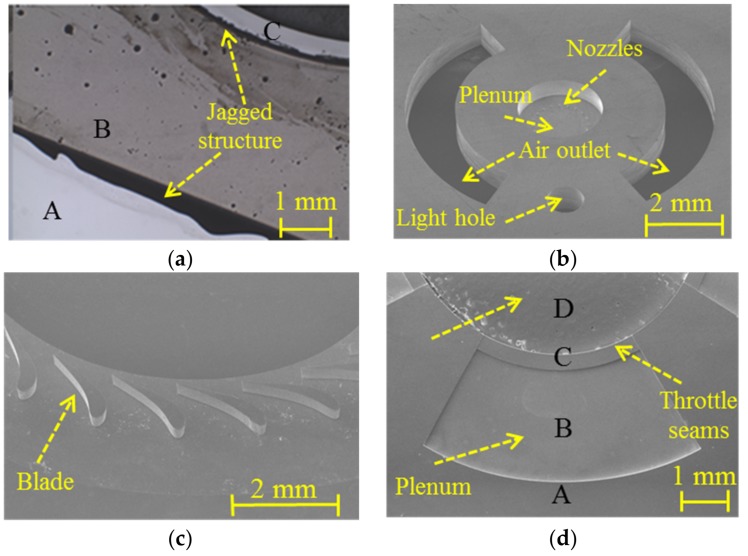
The etched multi-depth structures in MABs. (**a**) Photoresist masking method caused jagged structures; (**b**–**d**) Silica masking method achieved high-perpendicularity and integrated structures on forward side of L1 (**b**); forward side of L2 (**c**); and, aft side of L2 (**d**).

Two different methods were used to achieve multi-depth etching on the forward side of L1 layer and aft side of L2 layer. The etched structures were observed under a Nikon MM-400 microscope and SNE 4500 SEM. Figure 10a shows structures of L2 layer after the second coating using the traditional method. Jagged structure around the step that was caused by uneven coating could be found easily, which did not occur in the etched structures using the innovative method. High-accuracy depth control was achieved and the silicon micro pillars phenomenon did not appear at the bottom, which demonstrated the feasibility of multi-depth etching using both silica film and photoresist as DRIE mask. Etched structures with three depths in the forward side of the L1 layer and three depths in the aft side of L2 layer are shown in Figure 10b,d, respectively, which were consistent with the design structures. Figure 10c shows complete and uniform blades on the forward side of the L2 layer.

In this DRIE experiment, the thickness of the SPR220 photoresist was generally 6 μm, which was limited by the rotation speed of coating process. A structure with a depth of 90 μm was fabricated during the first DRIE etching, while at the second photoresist coating, the 6 μm-thick photoresist had to cross over the 90 μm-deep step, which would inevitably cause non-uniform coating and non-designed etching. Instead, when silica film was adopted as an etch mask using the innovative method, all of the coating processes were completed before DRIE etching. Therefore, the 6 μm-thick photoresist was just required to cross over 2 μm-thick silica film at the second coating, which was quite thinner than the 6 μm-thick photoresist and sufficient etching mask protection was provided. In addition, once the only oxidation process was completed before any fabrication process, no other oxidation processes need to be done, which will reduce warpage that arose from accumulation of residual stress during oxidation process.

It was found in this study that a silica film mask can not only be of great use to achieve etching for three-depth structure on the same side, but also be of great use in more-depth etching. Based on the above analysis, if the accurate etch selectivity is obtained and the thickness of silica film is precisely controlled, multi-depth etching using silica film as etching mask can be achieved.

### 3.3. Direct Bonding of Three Layers

An AWB-04 wafer bonder that was produced by AML Company was used for the bonding experiment. To improve bonding quality, the maximum warpage, and surface roughness of the original wafers were controlled strictly at less than 15 μm and 1 nm, respectively, which was realized by the wafer supplier. A comparative experiment of SDB was conducted using 12 wafers divided into four groups and the bonding quality was detected. The details regarding silica film protection and the etched structures are shown in Table 2. All of these process parameters were typical for this bonder and annealing system.

**Figure 11:**
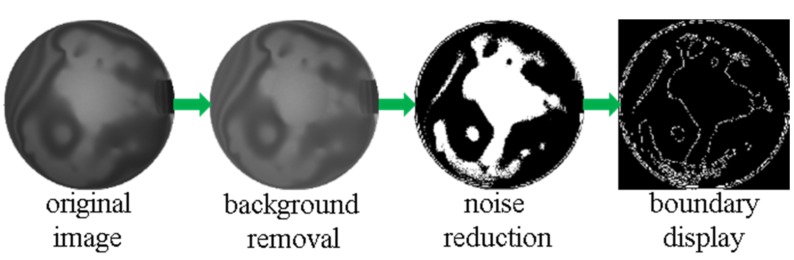
Image processing steps to calculate bonding rate.

Bonding quality was detected by infrared camera qualitatively and analyzed quantitatively by a MATLAB image processing program, shown in Figure 11. The original photo went through background removal, noise reduction, and eventually displayed the bonding boundary with edge function. Subsequent to the image process completion, the bonding rate defined as the ratio of bonded area to total area of the 4 inch wafer was calculated. As shown in the fourth photo of Figure 11, the bonding rate is equal to the area of the white part divided by the total area of both the black and white part.

**Figure 12:**
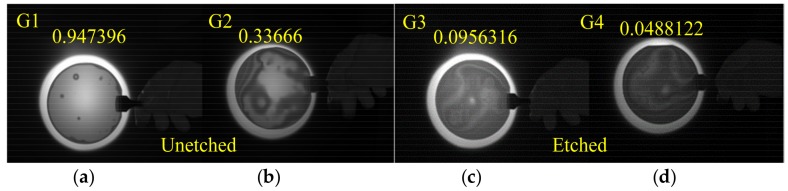
Images of different bonding results obtained by infrared camera. (**a**) Bonding results of unetched wafers with silica film; (**b**) Bonding results of unetched wafers without silica film; (**c**) Bonding results of etched wafers with silica film; (**d**) Bonding results of etched wafers without silica film.

The bonding results of the four groups are shown in Figure 12. Bonding rate of the wafers with the protection of silica film was closer to 94.7%, as shown in Figure 12a. There existed several obvious un-bonded point regions, which were thought to be contaminants due to the removal of protective silica film during the process when the wafer was transferred from the wet process station to the bonder. Figure 12b shows that the bonding rate of the unprotected wafer decelerated to 33.7%. The unetched wafers were double side polished and no oxide was grown on the surface. In the process of coating, cleaning, and microscopic observation, surface of wafers (without oxide) directly contacted with surface of glassware, microscope stage caused friction and scratches. Therefore, inevitable contamination, which caused a decrease in surface cleanliness and flatness, would affect the bonding rate. Figure 12c,d shows the bonding infrared image of etched wafers. Eight MAB devices with a size of 2 cm × 2 cm and four alignment markers were fabricated on a four-inch wafer and elevated structures led to the reduction of the available bonding area by 50%. Due to the etched structure, it was impossible for the three wafers to be bonded at every part of the wafers’ surface, and thus the bonding rate of 9.6% for a large etched structure was acceptable. The inevitable direct impurity contamination and rub injury to the wafer surface resulted in low cleanliness and increased roughness, which caused the bonding rate to drop from 9.6% to 4.9%. When compared to G1 and G2, the bonding rate of G3 and G4 decreased seriously even adding to the area of elevated structures, which was caused by a small warpage that arose from the accumulation of residual stress during the long etching process and an increase of the difficulty for the bonding wave [23] moving from center to edge.

**Figure 13:**
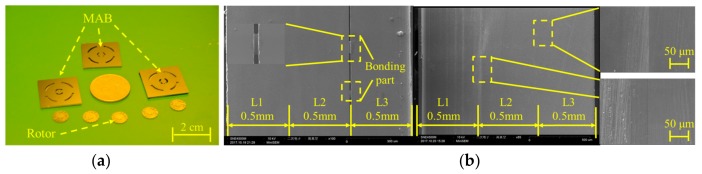
Diced MAB device and bonding status in the interface. (**a**) Photo of three-layer MABs and their micro rotor; and, (**b**) SEM image of bonding interface with poor and good bonding quality.

The wafer stack of G3 and G4 were carefully diced into 2 × 2 cm devices of MAB by a wafer cutting machine after three-layer SDB, shown as Figure 13a. Each build of the original MAB consists of an eight wafer stack on four inch wafers. Further observation of diced devices was carried out and the results are shown in Figure 13b. Five of the diced eight wafer devices of G3 showed no gap in the interface. However, a clear gap of 3 μm was observed near the interface in the other three devices of G3 and the whole devices of G4. Without silica film protection, the wafers in G4 had much poor bonding quality. In addition, the gaps observed in stacks of G3 all appeared between L1 layer and L2 layer, which were bonded after the bonding process of L3 layer and L2 layer. This phenomenon shows that the warpage of the silicon wafer has an important effect on multilayer bonding. Therefore, it is necessary to minimize the residual stress of the wafers in the previous process.

**Figure 14:**
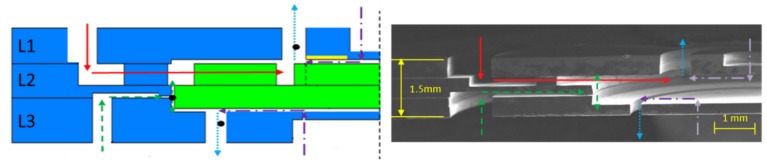
Cross-sectional view of designed MAB structure and manufactured MAB structure.

The cross-sectional view of designed structure and fabricated structure are shown in Figure 14. The results show that with innovative BOE etching, multi-depth etching and silica film protection assisted bonding methods, high-accuracy morphology control, and efficient three-layer SDB can be achieved in the manufacturing process of MABs.

## 4. Conclusions

The application of silica film in the manufacturing process of micro air bearing to realize multi-depth etching and efficient three-layer bonding was examined experimentally in this study. The mechanisms and improvement method of wet etching defects were also analyzed. A 2 × 2 × 1.5 cm silicon micro air bearing device has been manufactured with high morphology accuracy. The results indicate that:

1. Using SPR220 photoresist, which has better adhesion with silica film as a BOE etching mask that was supplemented with ultrasonic vibration to expel bubbles, can help to eliminate the undercut defect in the wet etching process and improve the uniformity and integrity of micro structures, especially in wet etching of a small-sized structure, like nozzles, spiral grooves, and turbine blade trailing edges.
2. Multi-depth etching with precise depth control can be realized by using both photoresist and silica as an etching mask by only one oxidation process at the beginning. When compared to the traditional method using photoresist as a mask, jagged etching structures could be avoided by using this innovative method and multi-depth etching can be achieved. Warpage caused by the residual stress was reduced compared to the similar method with 1–2 thermal oxidation during the whole process to achieve three depths structures.
3. Silica film protection has a critical impact on three-layer silicon direct bonding. The bonding rate can be increased by 50% with intact protection of silica film before bonding. Difficulty of bonding will rise sharply with the increase of the number of bonding layers because of the accumulation of residual stress in previous binding process. Therefore, compact structure design to reduce the number of layers and reduction of residual stress in pre-sequence processes were suggested to improve the bonding quality.

The process method about silica film described in this paper can be applied in many other micromechanical devices requiring complicated microfluidic interconnects and efficient bonding.

## Figures and Tables

**Table 1:** Depth details of the wafer after each etch.

Etched Times	A/μm		B/μm		C/μm		D/μm	
	Silica	Si	Silica	Si	Silica	Si	Silica	Si
The 1st BOE	0	0	2	0	0	0	2	0
The 2nd BOE	0	0	0	0	0.5	0	0	0
The 1st DRIE	0	0	0	0	0	0	0	150
The 2nd ICP	0.45	0	0	90	0.45	0	0	90
The 3rd BOE	1.05	0	0	0	1.05	0	0	0
The 3rd DRIE	0.05	0	0	10	0	10	0	10
Total depth	1.55	0	2	100	2	10	2	250

**Table 2:** Silica film protection and etched structure details of the wafers.

Group	Silica Film Protection	Etched Structure
1	√	×
2	×	×
3	√	√
4	×	√
